# Heart rate variability and emotion regulation in adults with eating disorders or obesity: a systematic review

**DOI:** 10.1192/bjo.2021.122

**Published:** 2021-06-18

**Authors:** Chloe Gilkinson, Ulrike Schmidt, Lucy Gallop, Michaela Flynn

**Affiliations:** 1 Institute of Psychiatry, Psychology and Neuroscience, King's College London; 2 South London and Maudsley NHS Foundation Trust, Department of Psychological Medicine, Institute of Psychiatry, Psychology and Neuroscience (IoPPN), King's College London; 3 NIHR Maudsley Biomedical Research Centre PhD student; 4 Research Student, Department of Psychological Medicine, Institute of Psychiatry

## Abstract

**Aims:**

Emotion regulation (ER) impairments are central trans-diagnostic phenomena across the spectrum of eating disorders (EDs) and obesity, where maladaptive eating behaviors act to suppress negative emotions. Self-report assessments are the most commonly used tools for assessing an individual's ER capacity, however, subjective self-reporting is limited by a tendency toward response bias and issues with common method variance. Prior empirical and theoretical research supports the use of heart rate variability (HRV) to objectively assess individual differences in ER capacity. Several studies have examined the association between HRV and ER in EDs and obesity. However, to date, no review synthesising the overall findings exists. This review aimed to summarise the empirical evidence that has examined the relationship between ER and HRV in adults with EDs/obesity, in addition to assessing the validity of HRV as a physiological biomarker of ER in these populations.

**Method:**

A comprehensive search was performed on PubMed, MEDLINE and PsycINFO, with identified studies screened against a priori inclusion/exclusion criteria. Eligible studies underwent quality-assessment using the Joanna Briggs Institute Critical Appraisal Tools, and data were synthesised qualitatively.

**Result:**

17 publications were included, consisting of data on participants with obesity, binge eating disorder (BED), bulimia nervosa (BN), anorexia nervosa (AN), and/or subclinical presentations. Studies were small (average sample size n = 46.4), predominantly female (87.9%), and were highly variable in methodology, with different diagnostic tools, self-report measures, and emotional tasks/paradigms used.

**Conclusion:**

The evidence suggests that HRV is a valid, objective biomarker of ER impairments in AN, BN, BED, emotional eating, and obesity. Despite some inconsistencies, likely attributable to methodological heterogeneity, EDs/obesity appear to be characterised by irregular resting state vagal activity and abnormal stress reactivity. Furthermore, the autonomic dysfunction observed across EDs/obesity may be reversible by novel effective interventions such as HRV-biofeedback or PlayMancer videogame therapy.

